# Acute arterial ischemia in COVID-19

**DOI:** 10.1590/1677-5449.210022

**Published:** 2021-06-16

**Authors:** Silvia Maqueda Ara, Marta Ballesteros Pomar, Nuria Sanz Pastor, Cristina Nogal Arias, Marcos Del Barrio Fernández

**Affiliations:** 1 Complejo Asistencial Universitario de León – CAULE, León, España.; 2 Universidad de Madrid, Madrid, España.

**Keywords:** COVID-19, ischemia, thrombosis, COVID-19, isquemia, trombose

## Abstract

Since the coronavirus pandemic set in in Spain in March 2020, a noteworthy increase in the incidence of acute limb ischemia (ALI) has been observed. It has been recently discovered that SARS-CoV 2 may lead to ALI secondary to arterial thrombosis. Elevation of D-dimer (DD) in patients with coronavirus infection (COVID-19) indicates that a hypercoagulable state causes acute arterial thrombosis. A remarkably high DD elevation has been reported to be a poor prognosis factor in COVID-19. The ways in which SARS-CoV 2 results in arterial thrombosis may be multiple. On the other hand, surgical revascularization for ALI is associated with poor outcomes in COVID-19 patients, probably in relation to hypercoagulability. Here, we describe two ALI cases in patients who required urgent surgical treatment for limb salvage and were positive for the novel coronavirus infection (COVID 19).

## Resumo

Desde que a pandemia pelo novo coronavírus se estabeleceu na Espanha, em março de 2020, um aumento notável da incidência de isquemia aguda de membros foi observado. Recentemente, descobriu-se que o coronavírus 2 causador da síndrome respiratória aguda grave (SARS-CoV-2) pode ocasionar isquemia aguda de membros secundária à trombose arterial. A elevação do D-dímero em pacientes acometidos pela doença do novo coronavírus (COVID-19) indica o estado de hipercoagulabilidade como causa da trombose arterial aguda. Vale destacar que a alta elevação do D-dímero foi relatada como um fator de prognóstico reservado na COVID-19. Há diversas maneiras pelas quais o SARS-CoV-2 pode resultar em trombose arterial. Em pacientes com COVID-19, a revascularização cirúrgica para isquemia aguda de membros está associada a desfechos desfavoráveis, provavelmente relacionados a hipercoagulabilidade. Descrevemos dois casos de isquemia aguda de membros de pacientes que necessitaram de tratamento cirúrgico de urgência para salvamento de membro e que haviam testado positivo para COVID-19.

## INTRODUCTION

Since the coronavirus pandemic set in in Spain in March 2020, a significant increase in the incidence of acute limb ischemia (ALI) has been observed. Here, we describe two ALI cases in patients with coronavirus infection (COVID-19).

## PATIENT 1

### Clinical situation

A 70-year-old woman presented to the emergency department with right lower limb pain and coldness for 3 days. The pain developed suddenly without associated trauma and became worse over time. No history of cough, fatigue or dyspnea was elicited; however, she had presented fever 9 days previously.

She had a medical history of diabetes mellitus and hyperlipidemia.

The patient was afebrile and in no respiratory distress; pulse oximetry of 94% on 4L of oxygen. The right leg looked pale from the level of the knee. There was loss of touch sensation in the foot and she had difficulty moving the leg. All right lower extremity pulses were absent. There were no arterial Doppler signals. Pulses in the left leg were normal.

Laboratory results are summarized in Table 1. They included leukocytosis (white blood cell count of 28,800) and elevated fibrinogen levels (642 mg/dL). D-dimer (DD) was markedly increased (72,016 ng/ml). Other results included Creatinine: 2.38mg/DL; aspartate aminotransferase (AST): 231U/L and alanine aminotransferase (ALT): 149U/L; creatinine kinase (CK): 11,427 IU/L; lactate dehydrogenase (LDH):669 IU/L; and C-reactive protein (CRP): 98.2 mg/l.

**Table 1:** Laboratory findings of COVID patients.

**LABORATORY FINDINGS**	**Patient 1**	**Patient 2**
White-cell count (per mm^3^)	28800	9200
Neutrophils (%)	81%	88%
Lymphocytes (%)	9%	8.7%
Monocytes (%)	7%	3.2%
Platelet count (per mm^3^)	382000	221000
Hemoglobin (g/liter)	12.3	14.5
Prothrombin time (PT) (sec)	15.2	21
Activated partial-thromboplastin time (APTT) (sec)	28.2	29.8
Aspartate aminotransferase (AST) (U/liter)	231	54
Alanine aminotransferase (ALT) (U/liter)	149	25
Creatinine (mg/dL)	2.38	0.74
Creatine kinase (U/liter)	11427	329
Lactate dehydrogenase (U/liter)	669	869
Fibrinogen (mg/dL)	642	767
D-dimer (ng/mL)	72016	245196*
High-sensitivity C-reactive protein (CRP) (mg/dL)	98.2	393
Serum ferritin (ng/mL)	623	674
IL-6 (pg/mL)	-	29.4

Computed tomography (CT) of the chest showed ground-glass opacity and pulmonary infiltration.

**Figure 1:**
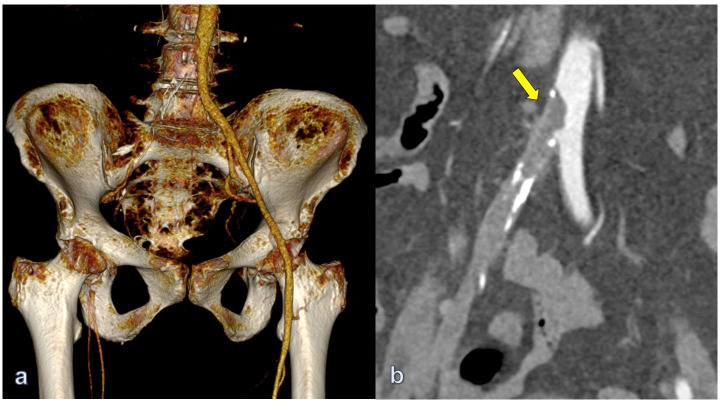
Preoperative computed tomography angiography (angio CT) with volume rendering 3D reconstruction: thrombotic occlusion of the right common and external iliac arteries with patent right femoral bifurcation. Of note, healthy and patent left Ilio-femoral axis (a). Iliac intraluminal thrombus protruding into aorta (arrow) (b) (patient #1).

**Figure 2:**
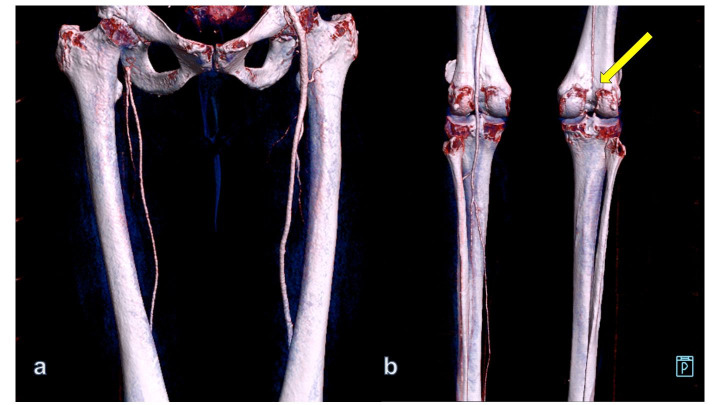
Angio CT with volume rendering 3D reconstruction showing infrafilling of a patent right superficial femoral artery (a). Total occlusion of the right popliteal artery (arrow) with absence of tibial vessels (posterior view). In the left lower extremity, all infrapopliteal vessels are patent (b), (patient #1).

The patient’s angio-CT scan demonstrated mural aortic thrombus at the infrarenal abdominal aorta as well as thrombotic occlusion of the right iliac artery and infrapopliteal vessels (Figures 1 and 2).

A COVID-19 nucleic acid detection test was negative.

### What was done

**Figure 3:**
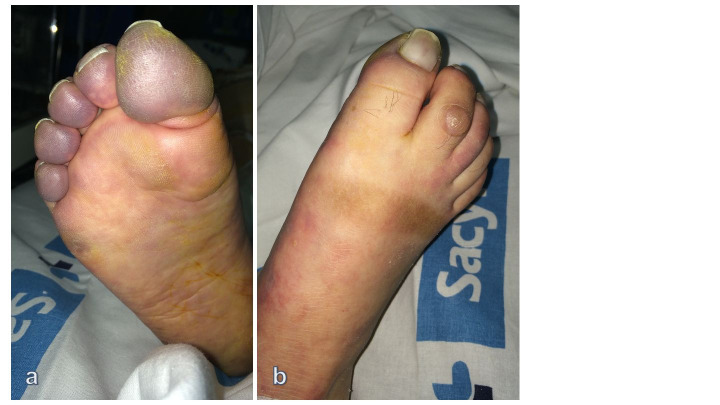
Picture showing patient’s foot several hours after revascularization surgery. Digital cyanosis and fixed staining in foot indicate failed revascularization (a, b) (patient #1).

The patient was taken to the operating room and underwent a femoral thrombectomy. By means of a Fogarty embolectomy catheter (Le Maitre®; Burlington, MA, USA), an extensive thrombus was retrieved from the iliac artery and femoropopliteal segment. The patient received systemic anticoagulation with subcutaneous enoxaparin sodium at 80 mg once a day. She recovered femoral and popliteal pulses but no distal pulses and several hours later mottled cyanosis appeared at the level of the foot (Figure 3).

Given the irreversible ischemia signs in the foot, a major amputation was carried out 48 hours later.

Serological testing revealed IgG-IgM antibodies to SARS-CoV-2.

## PATIENT 2

### Clinical situation

The patient was a 65-year-old man, with diabetes mellitus, hyperuricemia, chronic obstructive pulmonary disease, and atrial fibrillation on oral anticoagulant (edoxaban 60 mg once a day). He described a 2-day history of right lower limb pain and coldness.

His physical examination revealed a pale and cool right leg, with dependent distal rubor. He presented femoral pulse with audible arterial Doppler signals at popliteal and distal level. He did not show any impairment of sensitivity or motility.

Duplex scan examination showed occlusion of the right superficial femoral and popliteal arteries.

**Figure 4:**
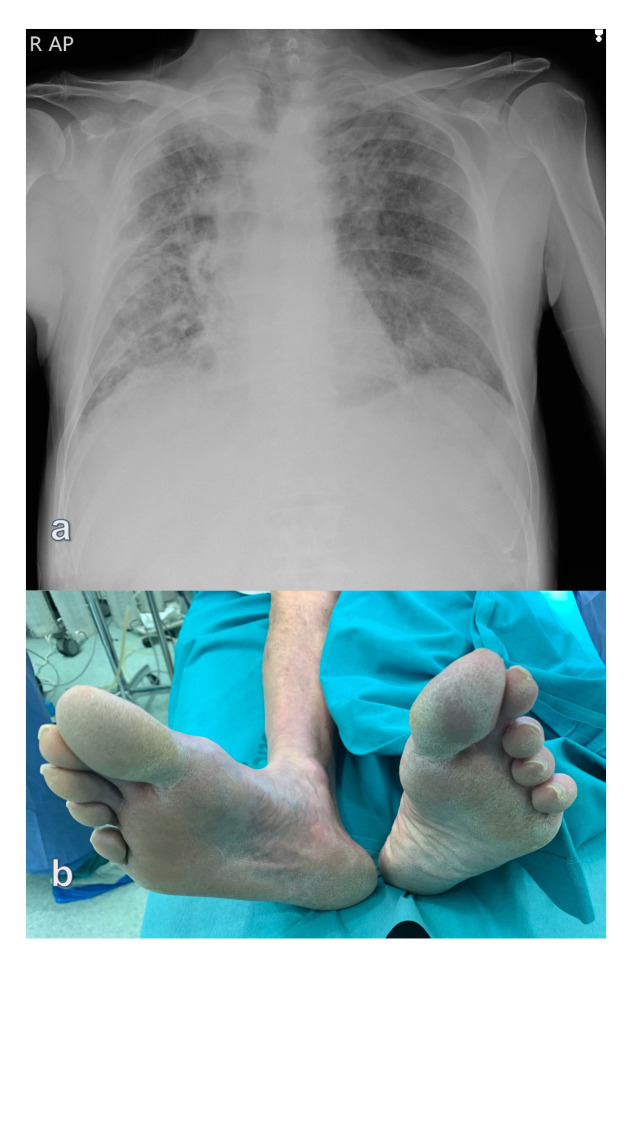
(a) Chest X-ray revealing bilateral peripheral ground-glass lung opacities (typical feature of COVID-19 pneumonia) as well as multifocal air-space disease; (b) Preoperative picture showing patient’s ischemic foot which required immediate revascularization (patient #2).

The patient denied any history of cough, fever, palpitation, or shortness of breath, but chest X-ray revealed peripheral ground-glass lung opacities and multifocal air-space disease (Figure 4a).

Laboratory results on admission are summarized in Table 1. The blood counts showed 9,200 white blood cells (88% neutrophils and 8.7% lymphocytes).

DD was extremely high (24.5196 ng/ml); CRP: 393 mg/L, CK: 329 IU/L, and LDH: 869 IU/L, all elevated.

The physical examination revealed irregular heart rate (102 beats/min), blood pressure of 133/81 mmHg, temperature of 35.9 °C, and oxygen saturation of 91%.

The COVID-19 nucleic acid detection test was negative. The following day, the patient’s state became aggravated despite active treatment. His right foot was cold and pale with cyanosis and paresthesia (Figure 4b). The patient noted difficulties in wiggling the toes. His right femoral pulse was faintly palpable and no distal Doppler signals were detected.

### What was done

The patient was taken to the operating room to perform an urgent thrombectomy. Subcutaneous enoxaparin sodium, 1 mg/kg every 12 hours was started. The clinical condition of the leg improved, but unfortunately the patient died 12 hours later. Serologic testing detected IgG-IgM antibodies to SARS-CoV-2.

## DISCUSSION

COVID-19 caused by SARS-CoV-2 has led to a pandemic, infecting over 3 million humans globally and causing over 240,000 deaths by early May of 2020.^1^

In addition to respiratory disease, SARS-CoV 2 infection may lead to thrombotic events. It has been reported that COVID-19 is associated with activation of the coagulation cascade, marked by increased DD levels.^1^^-^3

Recent guidance on management of coagulopathy in COVID-19 from the International Society of Thrombosis and Haemostasis (ISTH) arbitrarily defined markedly raised DD on admission as a threefold to fourfold increase.^1^ We found high DD values in both patients (higher than reported by others).^4^^-^6 This information could be useful, especially in patients with a negative COVID 19 nucleic acid detection requiring urgent surgery for ALI.

The ways in which SARS-CoV 2 leads to acute arterial thrombosis may be multiple:

First, virus infections are accompanied by an aggressive pro-inflammatory response and insufficient anti-inflammatory response. This might induce dysfunction of endothelial cells, resulting in excess thrombin generation.^1^

Second, release of pro-inflammatory cytokines, which are key mediators of atherosclerosis, may directly contribute to plaque rupture through local inflammation, induction of procoagulant factors, and hemodynamic changes.

Third, the hypoxia found in severe COVID-19 can increase blood viscosity and the hypoxia-inducible transcription factor-dependent signaling pathway.

Further studies are needed to understand how this new pathology leads to acute arterial thrombosis and if there is a way to prevent it.

## ETHICS COMMITTEE

This study was approved by the institutional research ethics committee. Authors declare that the manuscript is in accordance with the Helsinki Declaration and with local ethical guidelines. Patients were informed and signed informed consent.
